# Accuracy of photogrammetry, intraoral scanning, and conventional impression techniques for complete-arch implant rehabilitation: an in vitro comparative study

**DOI:** 10.1186/s12903-021-02005-0

**Published:** 2021-12-10

**Authors:** Bowen Ma, Xinxin Yue, Yujie Sun, Lingyan Peng, Wei Geng

**Affiliations:** 1 Department of Dental Implant Center, Beijing Stomatological Hospital, School of Stomatology, Capital Medical University, No. 4 Tian Tan Xi Li, Dongcheng District, Beijing, 100050 People’s Republic of China; 2 Department of Prosthodontics, Beijing Citident Stomatology Hospital, No. 109 North Xidan Street, Xicheng District, Beijing, 100032 People’s Republic of China

**Keywords:** Dental impression technique, Dental implants, Edentulous, Photogrammetry, Intraoral scanning, Conventional impression, Accuracy

## Abstract

**Background:**

To compare the accuracy of photogrammetry, intraoral scanning and conventional impression techniques for complete-arch implant rehabilitation.

**Methods:**

A master cast containing 6 implant abutment replicas was fabricated. Group PG: digital impressions were taken 10 times using a photogrammetry system; Group IOS: intraoral scanning was performed to fabricate 10 digital impressions; Group CNV: splinted open-tray impression technique was used to fabricate 10 definitive casts. The master cast and conventional definitive casts were digitized with a laboratory reference scanner. For all STL files obtained, scan bodies were converted to implant abutment replicas using a digital library. The accuracy of a digitizer was defined by 2 main parameters, trueness and precision. "Trueness" was used to describe the deviation between test files and reference file, and "precision" was used to describe the closeness between test files. Then, the trueness and precision of three impression techniques were evaluated and statistically compared (α = 0.05).

**Results:**

The median trueness was 24.45, 43.45 and 28.70 μm for group PG, IOS and CNV; Group PG gave more accurate trueness than group IOS (*P* < 0.001) and group CNV (*P* = 0.033), group CNV showed more accurate trueness than group IOS (*P* = 0.033). The median precision was 2.00, 36.00 and 29.40 μm for group PG, IOS and CNV; Group PG gave more accurate precision than group IOS (*P* < 0.001) and group CNV (*P* < 0.001), group CNV showed more accurate precision than IOS (*P* = 0.002).

**Conclusions:**

For complete-arch implant rehabilitation, the photogrammetry system showed the best accuracy of all the impression techniques evaluated, followed by the conventional impression technique, and the intraoral scanner provided the least accuracy.

## Background

It is generally believed that the passive fit of a prosthesis is a key factor affecting the long-term success of implant-fixed complete dental prostheses [1], and a compromised fit may cause a series of mechanical and biological complications [2]. Accurately recording implant locations is an integral prerequisite for fabricating an accurately fitting prosthesis, either by digital or conventional impression techniques.

In the workflow of conventional procedures, the splinted open-tray impression technique is mostly used to transfer the implant positions from the patient’s mouth through the impression material. The splined open-tray impression technique provides acceptable clinical results, but it requires complicated procedures that are time consuming and discomfortable for the patient. Moreover, the accuracy of definitive casts is influenced by multiple factors, for instance, impression materials [3], matching tolerance of components [4] and dimensional changes in master cast [5].

With the development of CAD/CAM, digital impression methods have gained popularity in implant dentistry. Intraoral scanning is a widely used digital impression technique in clinical practice. The accuracy of intraoral scanners used with implant-supported single crown and short-span restorations has been recognized [6–8]. However, whether intraoral scanners can be applied to complete-arch implant impressions is still questionable [9–17]. When scanning a complete edentulous arch, multiple clinical factors have been proven to influence the accuracy of intraoral scanning, such as intraoral scanner brand [18, 19], ambient light [20], scan body types [21–23], interimplant distance [11], scanning range [24], characteristics of the mucosa [25], movable mucosa [26], and scanning pattern [27].

Photogrammetry technology is a method of making precise measurements by using reference points in photographs [28–30]. As early as 1994, photogrammetry technology was introduced to implant dentistry to detect the marginal adaptation between the prosthesis and the implants [31]. In 1999, Jemt et al. [29]reported that photogrammetry technology could successfully record the implant replica positions of an edentulous mandible cast, and the accuracy of this technology was comparable to that of the conventional impression technique. However, due to its complicated operation, photogrammetry technology could not be further applied in clinical practice. With the development of digital technology, commercially available photogrammetry systems provide a new method for implant impression making for edentulous patients. Some case reports have reported that the photogrammetry system can be successfully used with complete-arch implant impressions with high framework fit [32–34]. However, current studies on the accuracy assessment of photogrammetry systems are very scarce, and the results are inconsistent [17, 35, 36]. Moreover, previous studies have not evaluated the position of implant abutment replicas but the position of scan bodies on the implants, which may not represent true clinical procedures.

The purpose of this study was to compare the accuracy of three impression techniques for complete arch implant rehabilitation: photogrammetry, intraoral scanning, and conventional impression techniques. Accuracy consists of trueness and precision (ISO 5725-1, DIN55350-13) [37]. Trueness was used to describe the deviation between test files and reference file, and precision was used to describe the closeness between test files. The null hypothesis was that no significant difference would be found in accuracy among the three different impression techniques.

## Methods

**Fig. 1:**
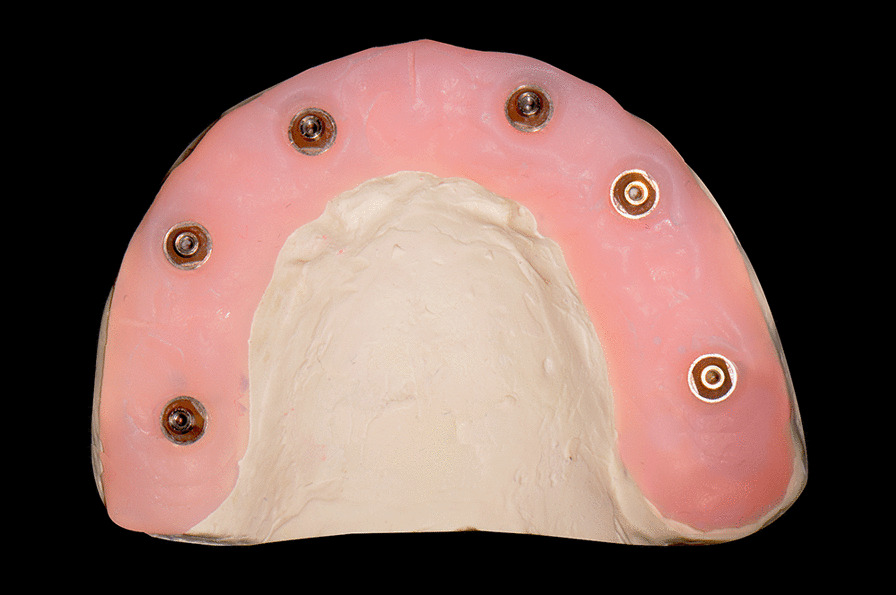
Maxillary completely edentulous master cast with 6 implant abutment replicas

A maxillary polymer resin model containing 6 implant abutment replicas (RC 4.6 mm repositionable analog for screw retained abutments; Institute Straumann AG) was prepared by using a polymer 3D printer (S300; UnionTech) and polymer resin (Model V2.0; UnionTech). Then, a stone master cast was fabricated from the polymer resin model by taking a splinted open-tray impression. The impression was poured with type IV dental stone (Marmoplast N; SILADENT Dr. Böhme & Schöps GmbH). This stone cast served as the master cast (Fig. 1). The depth and angulation of the implant abutment replicas are described in Table 1. From the master cast, three impression techniques were performed, namely, digital impression by using a photogrammetry system (group PG), digital impression by using an intraoral scanner (group IOS), and conventional impression (group CNV). The master cast was digitized using a laboratory reference scanner (E4; 3Shape; Software version 2.1.4.2) with an accuracy of 4 μm and exported to standard tessellation language (STL) file to serve as reference file. The laboratory reference scanner was calibrated prior to every scan.

**Table 1:** Position of implant abutment replicas of maxillary master cast

Implant tooth position	Depth	Angulation
Maxillary right lateral incisor	3 mm subgingival	0 degrees
Maxillary right first premolar	2 mm subgingival	7 degrees distal
Maxillary right first molar	1 mm subgingival	9 degrees distal
Maxillary left lateral incisor	2 mm subgingival	2 degrees distal
Maxillary left first premolar	2 mm subgingival	11 degrees distal
Maxillary left first molar	1 mm subgingival	7 degrees distal

**Fig. 2:**
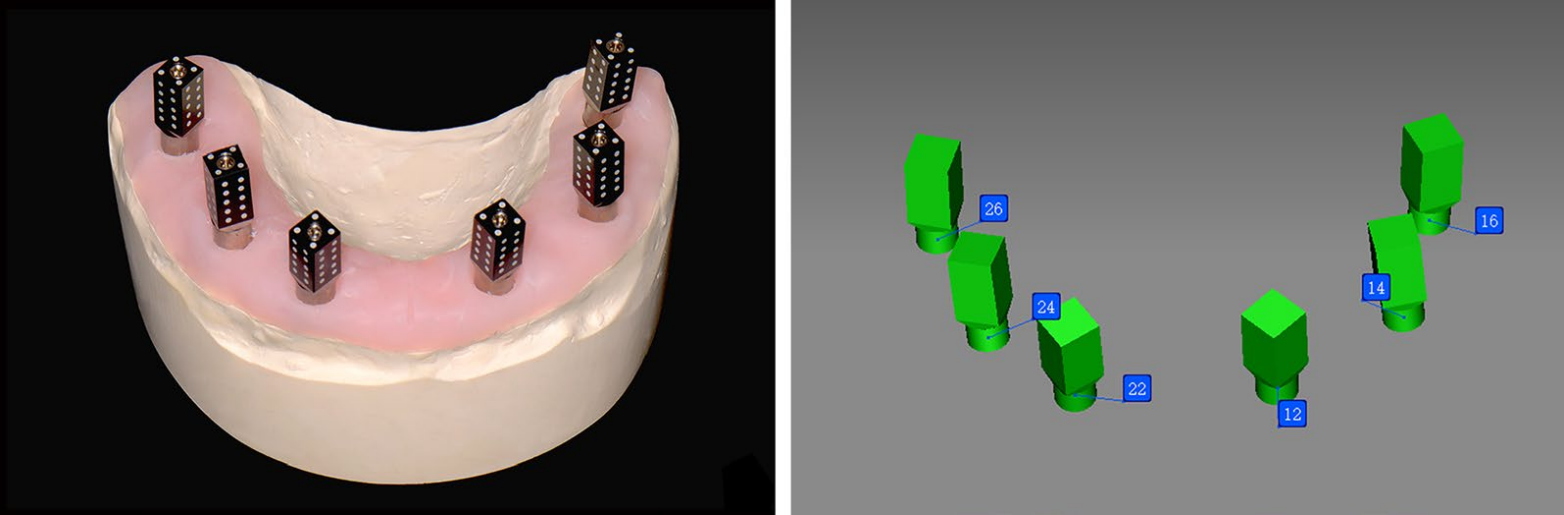
Digital impression procedures with photogrammetry system. **A** Scan bodies placed on implant abutment replicas of master cast before digitizing procedures. **B** STL file exported from the photogrammetry system

For group PG, scan bodies (ICamBody; Imetric4D Imaging Sàrl, Software version 9.1.79) were positioned and hand tightened on each implant abutment replication on the master cast (Fig. 2A), and a photogrammetry system (ICam4D; Imetric4D Imaging Sàrl) was used to digitize the master cast according to the manufacturer’s recommendations under room lightening conditions. The photogrammetry system was calibrated prior to every scan. The master cast was scanned ten times repeatedly without changing the position of scan bodies, and a total of 10 STL files were obtained (Fig. 2B).

**Fig. 3:**
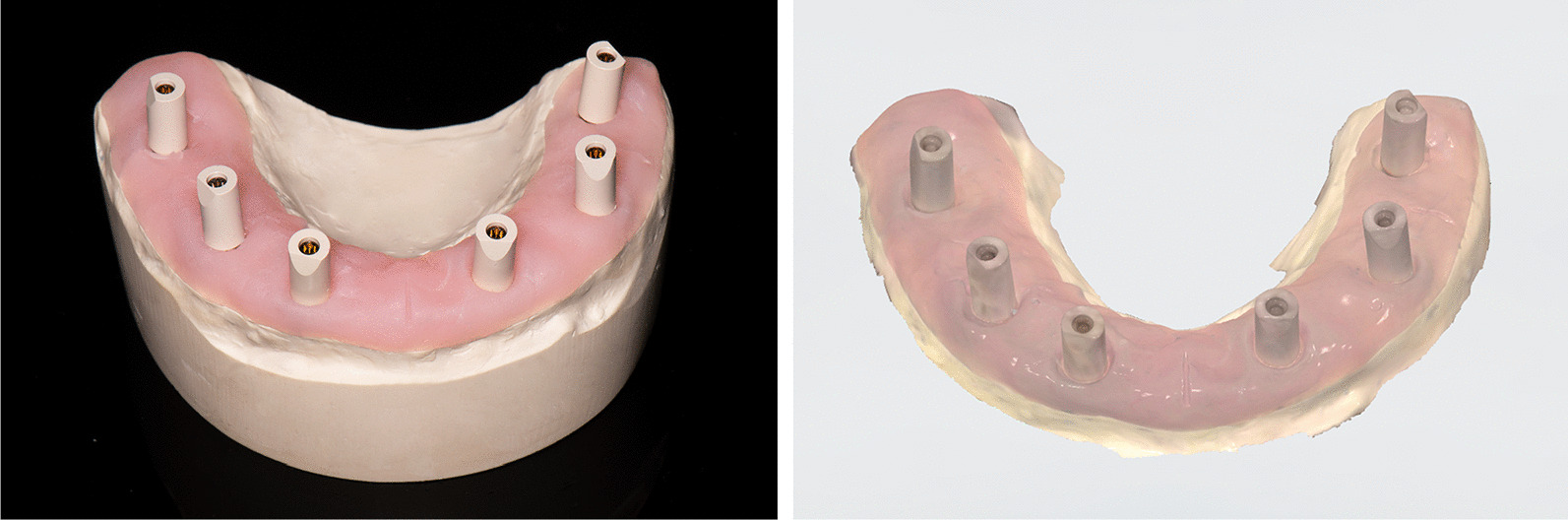
Digital impression procedures with intraoral scanner. **A** Scan bodies placed on implant abutment replicas of master cast before digitizing procedures. **B** STL file exported from the intraoral scanner

For group IOS, an intraoral scanner (TRIOS 3;3Shape; Software version 19.2.2) and scan bodies (CARES Mono Scan body for screw-retained abutment; Institute Straumann AG) were used to fabricate 10 digital impressions under the same room lightening conditions. All the scan bodies were brand new, and the intraoral scanner was calibrated prior to every scan. After scan bodies were screwed onto the implant abutment replicas on the master cast by hand tightening (Fig. 3A), the digital scan began from the occlusal surface of the scan body at the left molar area, continued to the contralateral right molar area, then went to the palatal surfaces of the scan bodies, and finally covered the buccal surfaces of the scan bodies. This scan pattern was in accordance with the manufacturer’s recommendations. The scanning was repeated ten times without changing the position of scan bodies, and a total of 10 STL files were obtained (Fig. 3B). The scanning procedures were performed by an operator with 5 years of clinical experience with intraoral scanning.

**Fig. 4:**
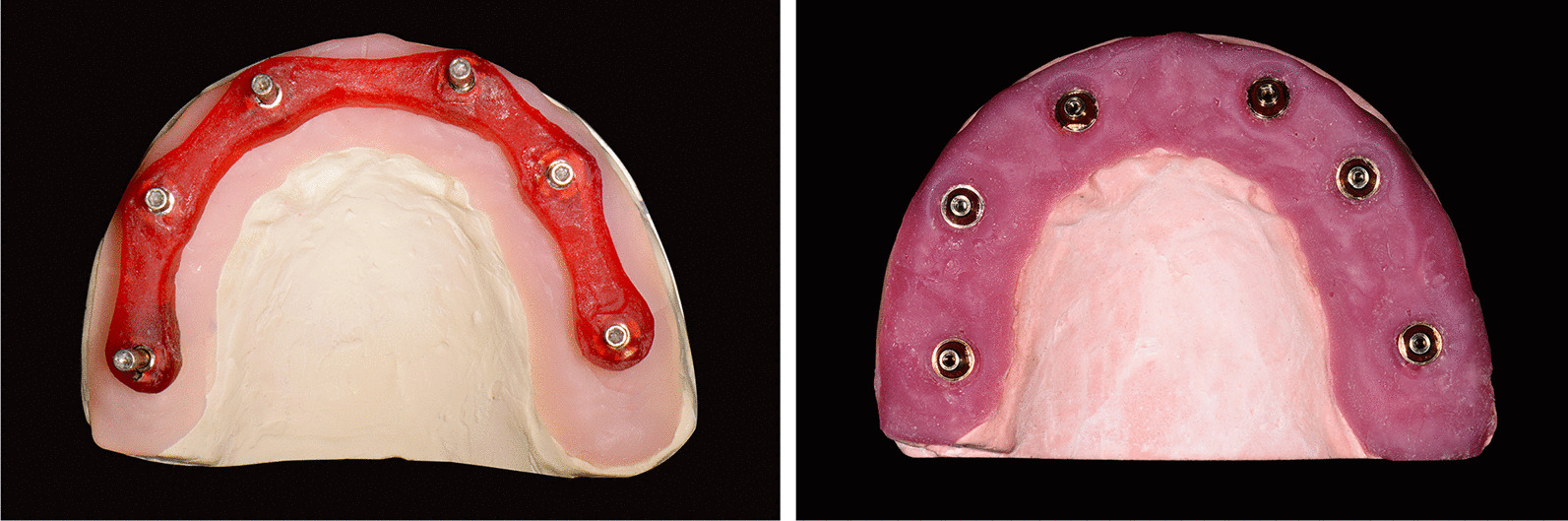
Conventional impression procedures. **A** Impression copings splinted with autopolymerizing acrylic resin. **B** Definitive cast

For group CNV, abutment-level impression copings (RC 4.6 mm impression coping for screw retained abutments; Institute Straumann AG) were connected to the implant abutment replicas on the master cast by hand tightening, and the impression copings were splinted using autopolymerized acrylic resin (Pattern Resin; GC). To reduce the polymerization shrinkage of the resin splint, the resin splint was sectioned and reconnected (Fig. 4A). A custom tray was fabricated using light-cured resin (LC-tray; Müller-Omicron GmbH & Co.KG). Tray adhesive (Tray adhesive; DMG) was applied 10 min before impression making, and the definitive impression was taken using the custom tray and polyether impression material (Impregum Penta Soft; 3 M ESPE). Impression procedures were performed in a room with a constant temperature range of 22–25℃. Four minutes later, impressions were removed from the master cast, and the implant abutment replicas were repositioned to the coping. The definitive cast was poured with Type IV dental stone (dentoststone 220; dentona AG) according to the manufacturer’s instructions (Fig. 4B). The definitive cast was digitized using a dental laboratory reference scanner (E4; 3Shape; Software version 2.1.4.2) with an accuracy of 4 μm and exported STL file. The conventional impression procedures were repeated 10 times to fabricate 10 definitive casts. Then, a dental laboratory reference scanner was used to digitize the 10 definitive casts, and a total of 10 STL files were obtained.

**Fig. 5:**
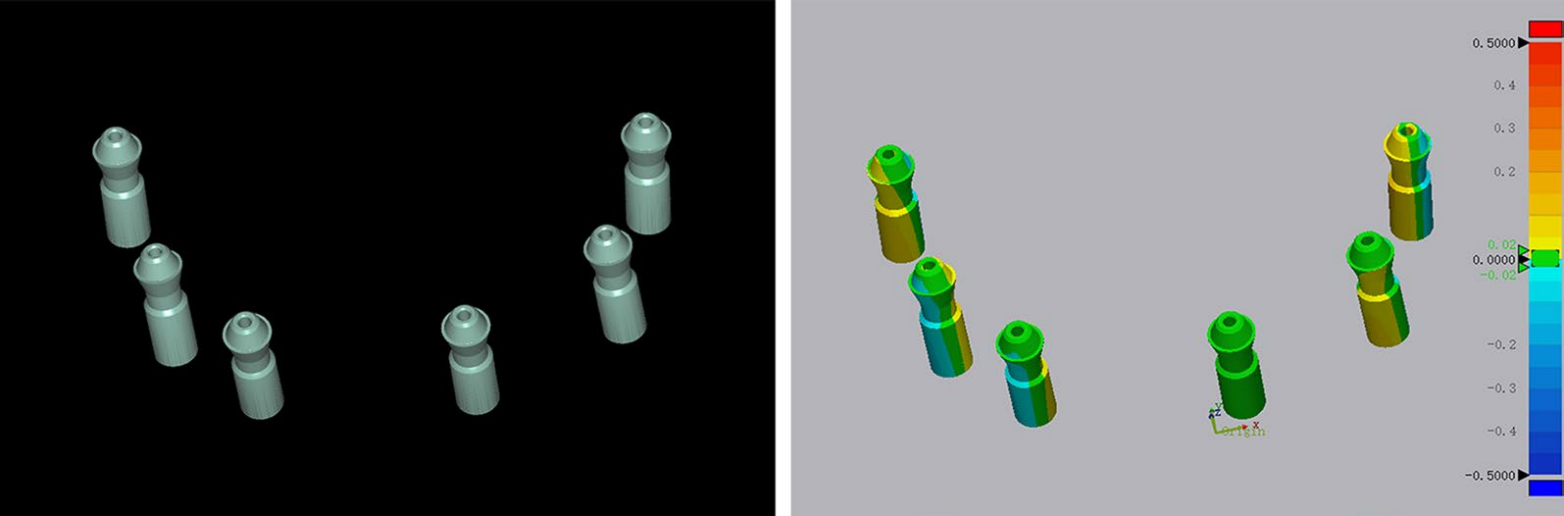
Evaluation of trueness and precision. **A** Scan bodies were converted to implant abutment replicas using a digital library. **B** Outcomes of 3D comparison are presented in the color maps, and RMS values were automatically calculated

All the STL files were imported to dental CAD software (exocad DentalCAD; exocad), and scan bodies were converted to implant abutment replicas using a digital library (Fig. 5A). [15] Then, updated STL files were imported to inspection software (Geomagic Control X; 3D systems) for trueness and precision assessments. The 2 STL files were superimposed using the “best fit algorithm”, and the three-dimensional discrepancy between 2 STL files was evaluated by the root mean square (RMS) error calculated by the inspection software. Then, a colorimetric map of the results was exported, and the surface tolerance of these deviations was chosen as 20 μm. (Fig. 5B). Trueness was evaluated by superimpositions and three-dimensional comparisons between reference file and test files, and a total of 10 RMS values were obtained in each group; precision was used to evaluate the three-dimensional deviation of the pairwise comparison of files within the test groups, and a total of 45 RMS values were obtained in each group [10, 16, 38, 39].

Statistical evaluation was performed using an analysis software program (IBM SPSS Statistics, v25; IBM Corp). The Shapiro–Wilk test revealed that the data were not normally distributed. Differences between groups in trueness and precision were evaluated using the Kruskal–Wallis test, and the Dunn–Bonferroni test was performed for post hoc analysis. The level of significance was set at α = 0.05.

## Results

The trueness and precision are shown by the median and interquartile range (IQR) of the RMS values (Tables 2, 3). The power test of the statistical analysis was greater than 80%. The Kruskal–Wallis test indicated significant differences for both trueness (*P* < 0.001) and precision (*P* < 0.001). Table 4 presents the results of post hoc analysis.

**Table 2:** Descriptive statistics of the RMS values for trueness (μm)

	PG	IOS	CNV
Median	24.45	43.45	28.70
Mean	24.43	43.78	29.75
IQR	0.73	6.17	7.90
SD	0.35	4.03	3.68

*PG* photogrammetry, *IOS* intraoral scanner, *CNV* conventional impression, *IQR* interquartile range, *SD* standard deviation

**Table 3:** Descriptive statistics of the RMS values for precision (μm)

	PG	IOS	CNV
Median	2.00	36.00	28.70
Mean	2.32	37.07	29.72
IQR	1.65	9.95	4.80
SD	0.85	3.98	7.85

*PG* photogrammetry, *IOS* intraoral scanner, *CNV* conventional impression, *IQR* interquartile range, *SD* standard deviation

**Table 4:** Comparison of trueness and precision among groups tested

	PG VS CNV	PG VS IOS	IOS VS CNV
Trueness	0.033*	< 0.001*	0.033*
Precision	< 0.001*	< 0.001*	0.002*

*PG* photogrammetry, *IOS* intraoral scanner, *CNV* conventional impression

*Indicated significant differences between groups (*P* < 0.05)

**Fig. 6:**
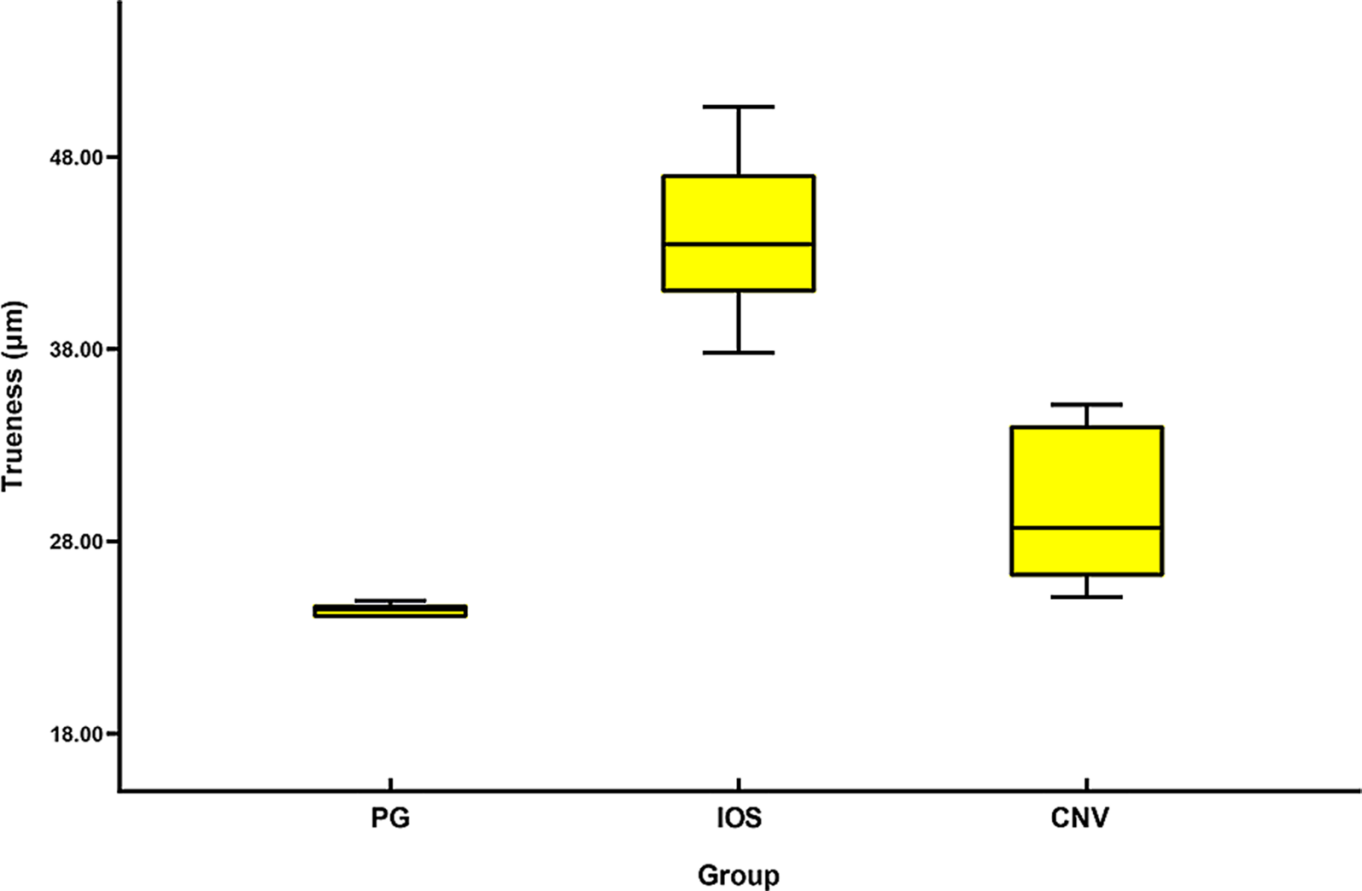
Median and interquartile range of trueness for group PG, IOS and CNV. PG, photogrammetry; IOS, intraoral scanner; CNV, conventional impression

The median of trueness was 24.45 (IQR 0.73), 43.45 (IQR 6.17), 28.70 (IQR 7.90) μm for group PG, IOS and CNV, respectively; Group PG gave more accurate trueness than group IOS (*P* < 0.001) and group CNV (*P* = 0.033), group CNV showed more accurate trueness than group IOS (*P* = 0.033). A boxplot of the trueness of the three impression techniques is shown in Fig. 6.

**Fig. 7:**
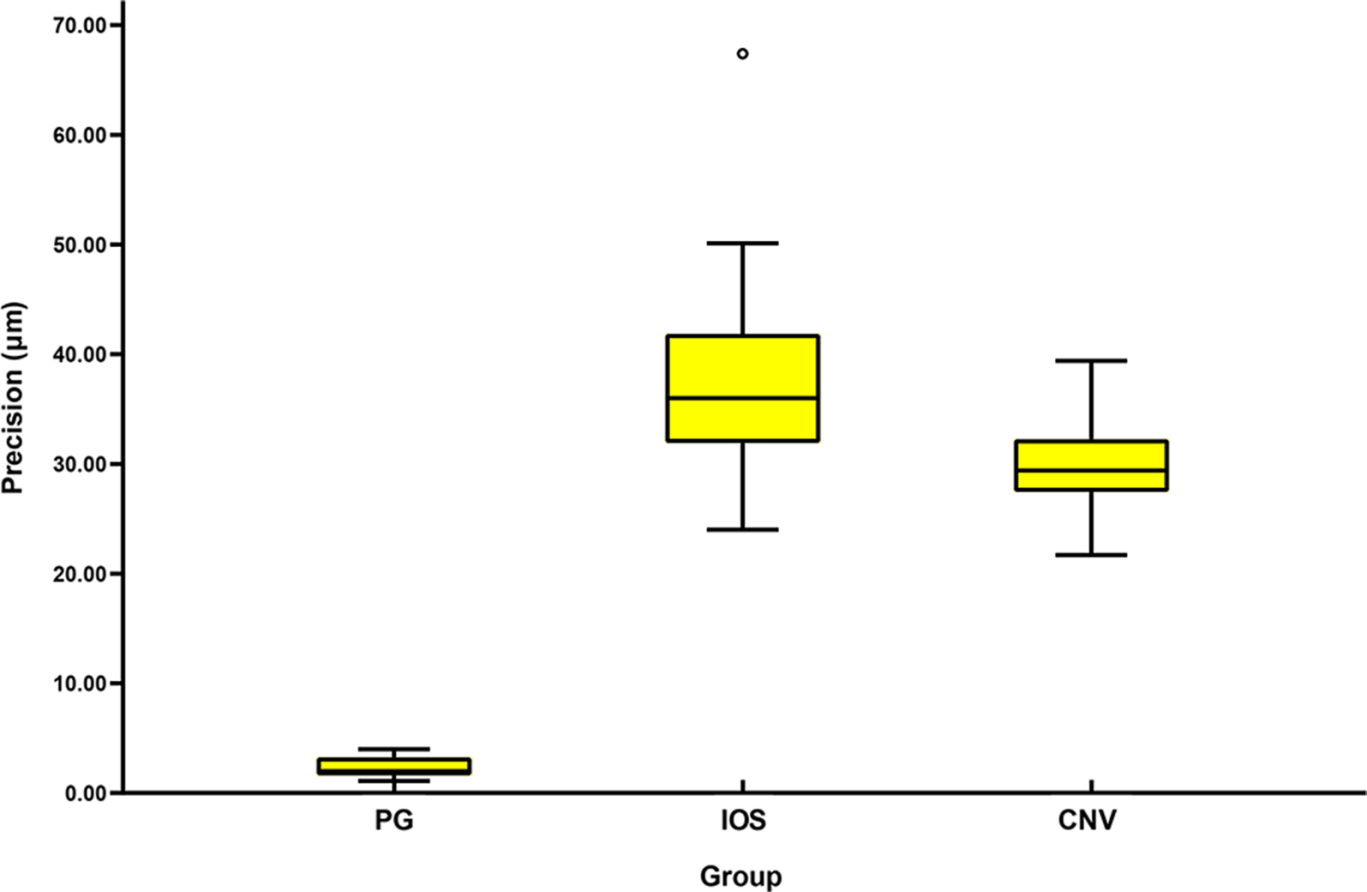
Median and interquartile range of precision for group PG, IOS and CNV. PG, photogrammetry; IOS, intraoral scanner; CNV, conventional impression

The median of precision was 2.00 (IQR 1.65), 36.00 (IQR 9.95), 29.40 (IQR 4.80) μm for group PG, IOS and CNV, respectively; group PG gave more accurate precision than group IOS (*P* < 0.001) and group CNV (*P* < 0.001), group CNV showed more accurate precision than group IOS (*P* = 0.002). A boxplot of the precision of the three impression techniques is shown in Fig. 7.

## Discussion

This study compared the accuracy of photogrammetry, intraoral scanning, and conventional impression techniques in an edentulous maxilla stone cast with 6 implant abutment replicas. The null hypothesis was rejected, as significant differences were found among the three test groups. For both trueness and precision, the photogrammetry system tested showed the best outcomes, the second place was the conventional impression technique, and the last was the intraoral scanner evaluated.

At present, research on the accuracy of photogrammetry systems is still very scarce, and the results are inconsistent. Tohme et al. [35] reported that the photogrammetry system exhibited better accuracy than intraoral scanner and conventional impression technique, which is consistent with the results of this study. However, Revilla-León et al. [17] came up with a different outcome with this study, compared with intraoral scanner and conventional impression technique, the photogrammetry system tested showed the least accuracy. Another study also reported by Revilla-León et al. [36] suggested that the photogrammetry system was less accurate than conventional impression technique. Comparing the 2 previous studies with opposite results, the different outcomes may be due to different study designs involving reference file and measurement methods. In previous studies, the reference file were obtained by a coordinate measuring machine, and then the linear and angular deviations were evaluated. In this study, the reference file were obtained by a laboratory reference scanner, and then the accuracy was assessed by root mean square error. Compared with laboratory reference scanner, coordinate measuring machine exhibit better accuracy and repeatability, but it is less accurate in accessing freedom plane, in addition to the size and shape of its spherical probe, it is impossible to detect complex and undercut area, which may influence the accuracy of the reference file, this is also the reason why the laboratory reference scanner was chosen in this study. As reported in multiple studies [18, 23, 40], 2 STL files were superimposed through the “best fit algorithm” in this study. The standard "best fit alignment" uses an iterative closest point (ICP) algorithm to align the STL files, which is not affected by the operator factors. The alignment is performed by minimizing the error between the distance of each corresponding data point [41]. The main limitation of this method is that inherent errors inevitably occur during the superposition process, which has a certain impact on the accuracy evaluation. This inaccuracy was avoided by using the root-mean-square error to measure 3D deviations, and RMS values offset the positive and negative deviations of the "best fit" between the reference file and test file. This kind of method has been used in many studies [10, 16, 38, 39].

Different studies have investigated the accuracy of intraoral scanners in complete arch implant rehabilitation, but there is no consensus. Some reports have shown that the accuracy of intraoral scanners can be comparable to that of conventional impression techniques [9, 10, 12–14, 17], whereas some studies have revealed that the conventional impression technique is still more accurate than intraoral scanners [11, 15, 16]. In this study, the RMS values of trueness and precision in group IOS were both significantly higher than those of the conventional impression technique, and the results indicated that intraoral scanning was still less accurate than the conventional impression technique. The possible explanation for this result is that the 3D images obtained by intraoral scanners are generated by a series of image stitches, a longer scanning path may lead to the accumulation of error, and the lack of a stable identification marker on the mucosal surface also influences the accuracy of intraoral scanning. Previous literature reports have proven that compared with partial dental arch scans, intraoral scans with larger scan areas have greater deviation [25, 42]. Another study [40] also suggested that the accuracy of the subsequent scan quadrant was lower than that of the first scan quadrant. Different techniques offering stable characteristics between implants have been described to facilitate intraoral scanning procedures. An in vivo study indicated that the use of an auxiliary geometric part significantly improved the accuracy of the intraoral scanning accuracy for implant-supported complete arch prostheses and facilitated the scanning process itself [43]. Another in vivo study also suggested that the extensional structure of the scan body could significantly improve scanning accuracy, but this in vitro study showed that the conventional impression technique is still more accurate than intraoral scanning [16]. After all, whether these techniques can actually improve the accuracy still needs to be further explored in vitro and in vivo.

The photogrammetry system overcomes the limitations of intraoral scanners in obtaining the location of implant abutments in complete-arch implant rehabilitation. Intraoral scanners generate 3D images by a series of image stitches, and a longer scanning path may lead to the expansion of error [25, 38, 42]. However, compared with the intraoral scanning technique, the photogrammetry system takes all measured data in each picture and generates director vectors of the exact position of the scan bodies in relation to one another with the help of reference points. This method makes it possible to calculate the locations of scan bodies without superimposing pictures, which potentially ensures greater accuracy. Additionally, the photogrammetry system has multiple cameras with a larger scanning range and faster scanning speed. The scanner acquires images outside the mouth, which minimizes the influence of saliva, blood and humid environments on accuracy. However, the photogrammetry system has certain limitations; it only records the position information of the implant abutments in the patient’s oral cavity. Therefore, other procedures are needed to obtain soft tissue information.

This study compared the three-dimensional position of implant abutment replicas, which was under the assumption that the accuracy of the implant abutment replica positions was more important than that of the peri-implant mucosa in complete-arch implant rehabilitation cases; therefore, the location of the scan bodies was converted to implant abutment replicas in the digital library. However, there is an inherent connection error between different components [4], and the location of the scan bodies may not represent the true position of the implant abutment replicas. However, connecting errors are clinically inevitable. Digital impression techniques require only 1 connecting procedure to obtain the location of implant abutments, while the conventional impression technique requires 2 connecting procedures.

There are still a few limitations to the present study. This in vitro study could not completely simulate a patient's oral situation. This in vitro study avoids the influence of oral saliva, gingival crevicular fluid, humid environment, mucosal mobility, and patient mouth opening. These advantages may also make the accuracy higher than the accuracy achieved with intraoral scanner or photogrammetry system in clinical applications. Further studies are needed to explore the accuracy of different photogrammetry systems, as well as the impact of the number of implants, interimplant distance, angle and depth on it. The present study provides a certain degree of support for the clinical application of photogrammetry system, but more in vivo and in vitro studies are needed to verify its effectiveness.

## Conclusions

Within the limitations of this in vitro study, the following conclusions were drawn:

1. The photogrammetry system obtained the lowest 3D discrepancy in terms of trueness and precision for the implant abutment positions.
2. The intraoral scanner tested resulted in the highest 3D discrepancy for both trueness and precision, representing the least accuracy among the three impression techniques tested.
3. The trueness and precision of conventional impression technique were both less accurate than photogrammetry system, but both more accurate than intraoral scanner.

## Data Availability

The data that support the findings of this study are available from the corresponding author upon reasonable request.

## Abbreviations

CAD: Computer-assisted-design
CAM: Computer-assisted-manufacturing
PG: Photogrammetry
IOS: Intraoral scanner
CNV: Conventional impression
RMS: Root mean square
STL: Standard triangulation language
ICP: Iterative closest point
IQR: Interquartile range
SD: Standard deviation
